# Chemical Impurities: An Epistemological Riddle with Serious Side Effects

**DOI:** 10.3390/ijerph17031030

**Published:** 2020-02-06

**Authors:** Ahmad Yaman Abdin, Prince Yeboah, Claus Jacob

**Affiliations:** Division of Bioorganic Chemistry, School of Pharmacy, Saarland University, D-66123 Saarbruecken, Germany; s8ahabdi@stud.uni-saarland.de (A.Y.A.); s8pryebo@stud.uni-saarland.de (P.Y.)

**Keywords:** epistemology, impurity, pharmaceutical, risk management, space of information, Xpurity

## Abstract

Chemical synthesis is a science and an art. Rooted in laboratory or large-scale manufacture, it results in certain side products, eventually compromising the integrity of the final products. Such “impurities” occur in small amounts and, within chemistry itself, are of little concern. In pharmacy, in contrast, impurities increase the potential for toxicity, side effects, and serious implications for human health and the environment. The pharmaceutical regulatory agencies have therefore developed regulatory and strategic systems to minimize the chemical presence or biological impact of such substances. Here, pharmaceuticals are turned from impure into more defined materials as part of a complex socio-technological system revolving around and constantly evolving its specific rules and regulations. Whilst modern analytical methods indicate the presence of impurities, the interpretations of corresponding results are gated by risk management and agreed thresholds. Ironically, this allows for entities with no identified chemical structures, and hence epistemologically outside chemistry, to be regulated in pharmaceutical products. We will refer to such substances which are not, epistemologically speaking, “chemicals” as Xpurities, in order to distinguish them from recognized and identified impurities. The presence of such Xpurities is surprisingly common and constitutes a major issue in pharmaceutical research and practice. We propose a Space of Information to deal with such impurities based on values regarding the presence, chemical identities, and biological activities. It is anticipated that this may enable pharmacists to handle such Xpurities more efficiently.

## 1. Introduction

During the last century, pharmacy and the pharmaceutical industry have developed at a truly breath-taking pace, from small pharmacies and dispensaries to multibillion-dollar global companies. Besides the famous research in and development of new drugs, commonly referred to as R&D, drug safety increasingly takes centre stage. Indeed, the last couple of decades have witnessed a fair number of pharmaceutical scandals, from insecure ingredients and incorrect dosage forms to intentionally fortified medications and accidental contaminations. Today, the prime focus in treating a medical condition is geared toward guaranteeing safety and comfort of and for the patient. This stipulates for high-quality medications and treatments accompanied by flawless manufacturing regulations and strategies.

Indeed, such efforts by healthcare authorities are conspicuous. Changing the acronym GMP for Good Manufacturing Practices, for instance, to cGMP, where the “c” stands for “current”, underlines the continuous commitment to comply with GMP [1]. Similarly, the United States Food and Drug Administration (USFDA) in 2004 announced the shift from Quality by Testing (QbT) to Quality by Design (QbD) [2]. This initiative was adopted successfully by the International Council for Harmonisation (ICH), thereby introducing several quality guidelines [3]. The main objective of QbD is to plan large-scale manufacturing processes based on the notion of generating high-quality products with little to no variation.

Nonetheless, the reality is more complicated as chemistry is always temperamental once products, side products, yields, and impurities are considered. In pharmacy, the control of such side products and impurities, which must be considered as risky contaminants, is therefore one of the major challenges facing the pharmaceutical industry and drug authorities today. Many previous and recent examples on the devastating impact of such manufacturing variation have been documented. The thalidomide scandal, for instance, remains to this day a subject of scientific, philosophical, social, and legal scrutiny [4,5,6]. This case in the 1960s raised immense awareness about the differences in biological activities of racemate compounds and shaped the current ethics of chemical synthesis. It inevitably influenced drug development processes and regulation. Another tragic case from the 1960s is the one of “Agent Orange” [7,8]. Although not directly connected to pharmacy and pharmaceutical manufacture, this case includes a synthetic chemical which, due to the accompanying dioxin impurity, disastrously affected millions of people and the environment of an entire country, namely Vietnam.

Since then, the analytical tools available to detect, identify, and subsequently minimize or eliminate such impurities have increased in number and sophistication. Nonetheless, such scandals continue. Very recently, the hypertension medication Valsartan hit the news worldwide. In July 2018, the European Medicine Agency (EMA) and the USFDA both issued recalls for certain batches of this drug due to a possible contamination with the carcinogenic compound *N*-Nitrosodimethylamine (NDMA). Two months later, the Chinese manufacturer Zhejiang Huahai Pharmaceutical Co. announced that these batches were additionally contaminated with another carcinogenic impurity, *N*-Nitrosodiethylamine (NDEA) [9,10]. The year 2019 also had its share of impurity-related issues in pharmacy, as tests indicated that some batches of the widely used H_2_-inhibitor Ranitidine also contained the already mentioned NDMA [11,12].

**Figure 1:**
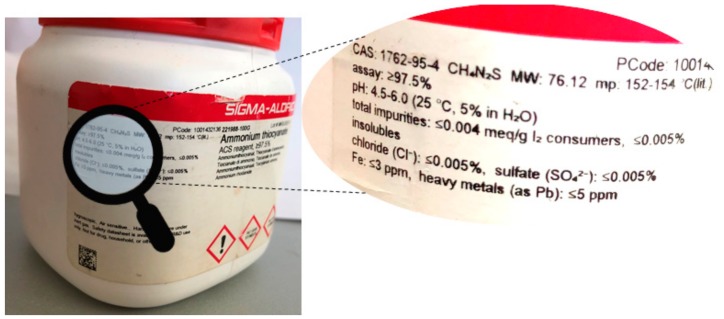
A commercial sample of ACS reagent ammonium thiocyanate with ≥ 97.5% purity. The remaining 2.5% mass is composed of various impurities is shown on the side of the container or listed in typical data sheets accompanying such a sample.

So, what exactly is the problem here? Is it because of inadequate analytical methods, or is there a deeper reason why such impurities occur and subsequently escape detection? This question provides room for discussing the philosophical implications of impurities. As we will notice, most compounds contain some impurities, and these are usually established and quantified. In practice, such chemicals contain the relevant information about their impurities on the box or bottle, as shown in Figure 1. These impurities are characterized and expected. In sharp contrast, there are also “hidden” impurities, that is, substances present which manifest themselves because of mass balance or as unidentifiable signals in certain spectra. These substances fall into two distinct categories. On one side, there are chemicals which are established and characterized, such as NDMA and NDEA, although they are not expected and hence not analysed for. On the other side, any synthesis may also produce entirely novel molecules, which have not been isolated or characterized chemically before, as has been the case with the impurity in Agent Orange. As such “novel” impurities are structurally unidentified, i.e., “outside the realm of chemical information”, they cannot be chased or tracked down that easily, as their analytical properties are also undetermined at that specific point.

In this study, we will refer to such an unidentified impurity as “Xpurity” to distinguish it clearly from impurities associated with an established analytics and chemistry. As will be discussed and illustrated with a mathematical model, an Xpurity is an entity which epistemologically falls outside the realm of current chemical information as it is unidentified with regard to its chemical composition and structure; curiously, it still does not fall outside the pharmaceutical product itself. Please note that Xpurities belong to the group of impurities, although the term “impurity” is broad and, in the guidelines, includes identified and unidentified entities. Therefore, coining a new term such as “Xpurity” and emphasizing the fact that unidentified entities are deliberately regulated in drug products is of considerable importance.

The following section will therefore provide a brief review on manufacture-related impurities in pharmaceuticals, their origins, techniques for detection, and characterization. The subsequent section will shed light on the regulations governing impurities in developed countries and the qualification of new chemical entities. In the fourth section, our philosophical analysis of impurities will provide new insights into the understanding and handling of impurities in general and Xpurities in particular. Here, the categorization of impurities follows the information of their presence, chemical identities, and biological activities rather than their synthesis and regulation by authorities.

## 2. There Are Always Impurities

In chemistry, the expression “impurity” refers to a chemical substance inside a confined chemical phase which differs from the chemical composition of that phase [13]. In order to assign a chemical substance the property of being “pure”, it has to fulfil three main criteria [14]. Firstly, a pure chemical should appear thermodynamically in at least one chemical phase and can also be characterized by its one-component-phase diagram. Secondly, practically speaking, a pure chemical should prove to be homogeneous (i.e., will show no change of properties after undergoing a wide variety of consecutive analytical chemical procedures). The perfect pure chemical will pass all attempts and tests of further separation and purification. Thirdly, and here we focus on the common chemical definition, it should not contain any trace of any other kind of chemical species. In reality, there are no absolutely 100% pure chemical compounds, as there is always some minute contamination. Indeed, as detection limits in analytical chemistry decrease, the number of impurities detected tends to increase [15].

Although impurities are considered a nuisance in chemical synthesis, they are generally of little concern as long as their identity is clear and their amounts are under control. Indeed, as shown in Figure 1, chemists may accept compounds with purities of 97.5% or even below as long as these reagents serve their purpose. From a pharmaceutical point of view, this matter is considerably more complicated. Here, the outcome of the chemical synthesis is not just a chemical substance per se, it is rather designed and manufactured to treat a specific medical indication. Here, impurities matter especially, as they may—unintentionally—be administered to patients together with the substance they are present in.

It is therefore hardly surprising that pharmacy has a specific *faiblesse* for this topic. The United States Pharmacopeia defines impurities as “any component of a drug substance which is not the chemical entity defined as the drug substance; for a drug product, any component that is not a formulation ingredient” [16]. The International Council for Harmonization (ICH) and collaborating agencies, which will be discussed in the next section, have adopted a similar definition [17].

Impurities are inevitable and can arise during the different stages of formulation, starting from raw materials, such as active pharmaceutical ingredients (APIs) and excipients. In the majority of cases, for instance, during the manufacture of APIs, the presence of impurities may be anticipated and subsequently mitigated. This is due to a combination of regulatory guidelines described as “conservative” and enormous efforts from the pharmaceutical industry to comply with these guidelines [18]. Still, the manufacture of APIs usually involves highly reactive reagents, increasing the probability of hazardous residues [19]. This places a heavy burden of responsibility on the pharmaceutical industry to achieve and deliver safe and efficacious drug products [20].

Identifying and characterizing a certain impurity can be accomplished through different methods, depending largely on the amount of the impurity and its sensitivity to the analytical method applied [21]. Here, it is common to carry out a series of different analyses to achieve a sufficient characterization of the sample and identification of all hints for an impurity found in the sample, such as a spot on the thin layer chromatography (TLC) or a peak in the mass spectrum, often with the assistance of modern and automated methods of characterization. These methods have the advantage of separating and quantifying impurities simultaneously, hence fulfilling their analytical goal. Nevertheless, the more classical methods, such as titration and colorimetry, have not lost any of their value either. Table 1 provides a brief and necessarily incomplete overview of some of the most commonly employed analytical methods in the pharmaceutical industry [15].

To exemplify the complexity of such procedures, we mention the effort of a team of researchers from Alkaloid AD Skopje, Macedonia, to identify and characterize Impurity RRT 0.95 [22]. In this case, the selective beta_1_ blocker Bisoprolol was contaminated with an unidentified impurity referred to as Impurity RRT 0.95. After performing a force degradation study to yield sufficient amounts of the impurity, the researchers subsequently analysed the yield with mass spectrometry, ^1^H-NMR and ^13^C-NMR spectroscopy, and ^31^P-NMR spectroscopy. The hitherto unidentified impurity was identified as a phosphomonoester of Bisoprolol resulting from an interaction between the API and the excipient calcium hydrogen phosphate. Impurities such as RRT 0.95 vary widely in their character, not only chemically, but also epistemologically. This aspect will form a major part of the subsequent discussion. As impurities are facet-rich and unavoidable, the next section will consider some of the strategies for coping with them, for instance, the analytical methods employed to guarantee the highest degree of purity in drug products and the regulations governing these impurities.

## 3. Regulatory Guidance on Impurities: Scope for Xpurities

Ethical, economic, and competitive reasons—and also safety and efficacy—require a tight control of impurities in drug products. This has resulted in a global effort to yield a better understanding of the concept of impurities in such drug products [15,23]. The effort devoted to such small quantities of chemical contaminants shows how vital and important these contaminants are.

Monitoring impurities, whether in the pharmaceutical industry or in research laboratories, may be affected by many requirements of compliance. A pharmacopeia or reference book, for instance, often provides the primary guidance for monitoring and regulating impurities [24]. Nonetheless, some tragic events in the history of the pharmaceutical industry demand a wider, possibly global collaborative effort to set governing guidelines within the industry.

The first such initiative was taken in the 1980s by the European Community, the European Union (EU) of today, laying the groundwork for harmonizing pharmaceutical regulatory requirements. In 1989, action plans for international harmonization were set up at the WHO Conference of Drug Regulatory Authorities in Paris. Soon afterwards, in 1990, the International Conference on Harmonization of Technical Requirements for Registration of Pharmaceuticals for Human Use (ICH) was founded at a meeting in Brussels hosted by the European Federation of Pharmaceutical Industries and Associations [3]. As mentioned in the Introduction, this institution today is referred to as the International Council for Harmonization and represents a collective effort of industry regulators and representatives from the EU, Japan, and the United States.

The ICH has been active for several decades now to develop guidelines on the characteristics for qualifying a new chemical entity to be considered as a drug compound and later a drug product which may be administered to humans for a specific medical condition. The guidelines prepared under the sponsorship of the ICH, whose main goal is to guarantee that there are consistent requirements for new drug applications, have been endorsed by the EMA and the USFDA [25,26].

**Figure 2:**
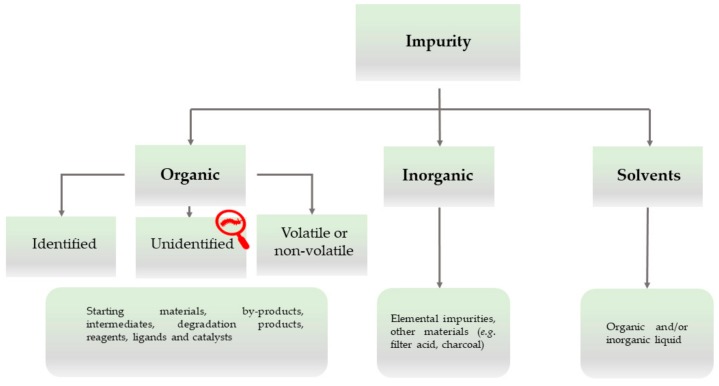
The classification of impurities by the ICH.

Impurities have always represented a major area of interest for the ICH and a considerable amount of information has been generated due to its efforts [27]. Various technological improvements and developments, together with the proper utilization of such information, have successfully achieved narrower limits for impurities and enforced more rigid controls on the production of drug substances and products. A unified industrial terminology and categorization for impurities are also a concern of the ICH, and some of this information, which is central to our subsequent epistemological discussion, is provided in Figure 2 [17].

The categorization of impurities by the ICH is broad and covers most kinds of potential impurities which may contaminate specific drug products and should be detectable and indeed detected with an appropriate chemical analytical method, often relying on reference standards and investigational strategies, such as the Technique-Oriented Strategy and the Chemically Guided Strategy, if and when the impurity profile of a chemical substance is in question [15]. Notably, this classification also provides scope for impurities which linger undetected or undetectable by the methods available, although they manifest themselves, for instance, through the mass balance, spots on plates, signals in spectra, or, in extreme cases, a toxic biological activity. These impurities are designated as “unidentified” and may include established chemicals prone in principle to detection if one is looking for them, and the Xpurities escaping any such attempt because of their undefined analytical profile(s).

**Figure 3:**
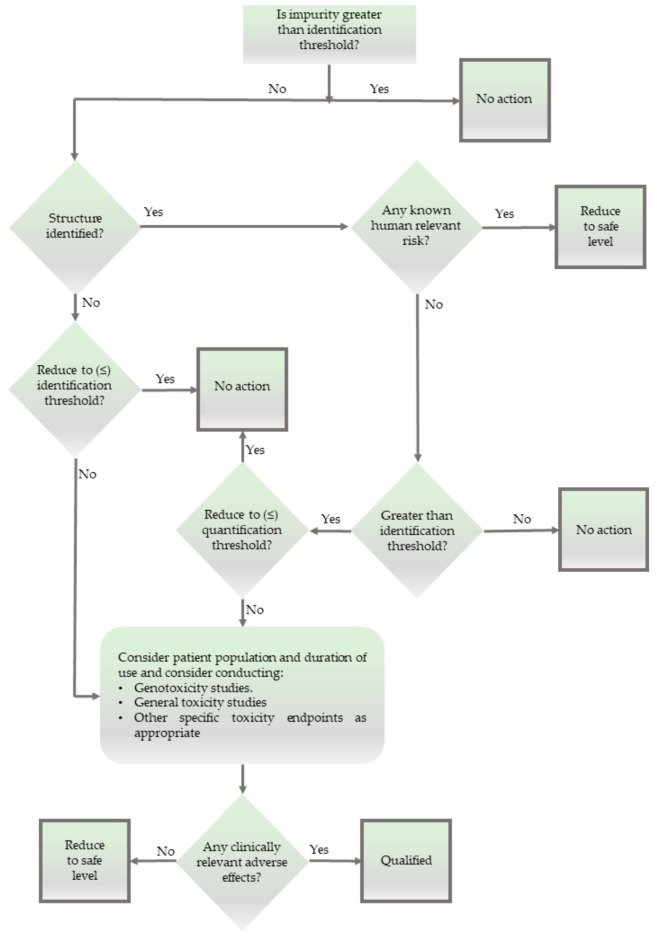
Flowchart of the ICH to identify and deal with impurities in medicines, adequately entitled “The Decision Tree for Identification and Qualification”. Please note that this flowchart explicitly provides scope for impurities which have no identified structure (i.e., impurities which are outside chemical analysis and formalism).

From a philosophical point of view, this subcategory of unidentified impurities is therefore of special interest, as these impurities differ from identified and structurally established impurities. The glossary attached to the ICH classification in Figure 2 and Figure 3 defines unidentified impurities as “an impurity for which a structural characterisation has not been achieved and that is defined solely by qualitative analytical properties (e.g., chromatographic retention time)” [17]. Indeed, impurity RRT 0.95 in Bisoprolol initially may have been found in this category before its chemical identity as a phosphomonoester of Bisoprolol had been determined. From the perspective of epistemology, these substances would be outside the scope of current chemical information. They would, strictly speaking, not be identifiable per se. We will illustrate this epistemological difference via a trivial example, i.e., cooking salt. In cooking salt, the potassium chloride naturally present is a household chemical, named sylvite, and is usually identified and even quantified, albeit not removed. In this respect, sylvite differs from impurities such as rubidium and caesium chloride, which may also be present in cooking salt in minute amounts, although they have not been analysed and determined for practical reasons (i.e., because their amounts are generally small and they are not toxic or otherwise worth specific attention). In contrast to these alkaline chlorides, Xpurities would be substances whose chemical identity (i.e., structure, formula composition, and analytics) and biological activity would be entirely undefined. Sea salt, for instance, may become contaminated with algae and some of their chemically complicated secondary metabolites may subsequently contaminate the salt. Such metabolites are difficult to detect by analysis as they may not be actively looked for in the cooking salt sample or as their analytical profile may be undefined altogether. Recently, researchers have taken a closer look at sea salt and found a spectrum of microplastics, that is, impurities which are in part established chemicals, and in part Xpurities [28].

Similarly, the hair you notice in your German sausage soup represents an identified impurity, the hair in your soup that you do not notice is unidentified in the soup, albeit not unidentified in general, and the funny-looking caterpillar that just dropped into your soup from the birch tree above, and that neither you have nor any entomologist has met before, must be considered a hitherto undiscovered species, an Xpurity. Swallowing one of the hairs will be harmless. In contrast, swallowing the caterpillar may upset your digestive system and also the entomologists, as you would probably be in breach of the Convention on Biological Diversity [29].

The limitations in analysis, opening the door for such Xpurities, are not only of analytical, pharmaceutical, and epistemological concern. They are also of ethical relevance, as unidentified impurities represent a *ticking Zeitbombe*. Is it therefore ethical to allow the presence of chemical substances in drug products without any information about their chemistry or biological activity, as suggested by the decision tree in Figure 3?

## 4. The Philosophical Analysis of Pharmaceutical Impurities: The Space of Information

Pharmaceutical impurities carry a certain ethical attribute, since they are chemicals with biological activity present in human drug products. Such impurities hardly promote any therapeutic benefits; they rather instigate harmful biological activities (e.g., toxic, carcinogenic, teratogenic, etc.). The biological activity of such substances is therefore of special interest, not only from a pharmaceutical, but, once again, also an epistemological perspective. In other words, how can we obtain biological information about an Xpurity which at this point is not captured by chemistry, albeit it is clearly present in the drug itself? Here, various aspects of information collide in a complex space containing information about the presence, that is, “existence” of the impurity, its “chemistry”, and also its “biology”, all under the presumption that the impurity is actually present at all.

As this may sound slightly confusing, we will briefly return to the rather tasty example of the caterpillar. The first question is, of course, whether the caterpillar is really in the soup. This is a metaphysical question about “presence”, not an epistemological one. This is followed swiftly by the question of whether we are actually aware that the caterpillar is in the soup, which indeed is now an epistemological issue. This is followed by the question of whether we can identify the caterpillar, for instance, as a tasty regional German culinary specialty or not. Turning toward biological impact, we then need to answer if the caterpillar is indeed tasty or possibly toxic if consumed.

**Figure 4:**
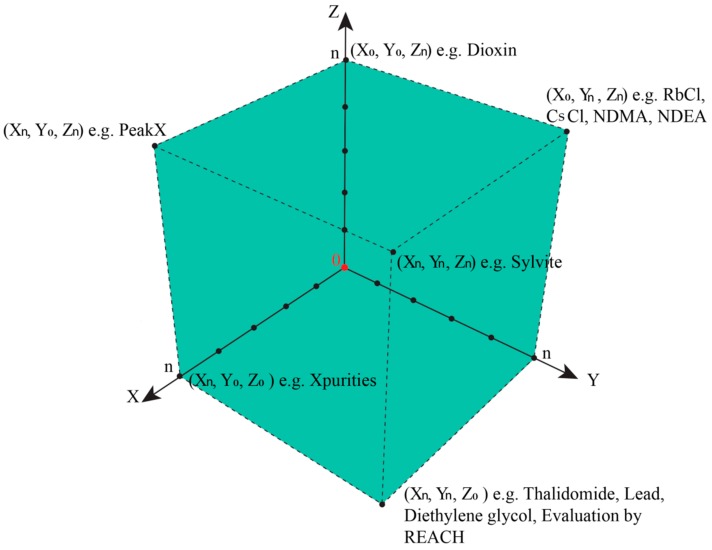
The Space of Information available for an impurity in a drug at a specific time. The different axes represent the relevant categories of information required for an impurity so the drug can be considered as safe. Each impurity may be placed in this space in order to estimate which kind of actions are required, for instance, closer inspection of the presence, chemistry, or biological activity, or no action at all. The Space also enables the construction of historical trajectories of impurities, as information about them increases. Please note that this Space may also be expanded in response to stricter regulatory requirements.

In such cases, the epistemological interdependencies between these different categories describing and classifying such impurities result in a complex landscape of information best represented by a Cartesian coordinate system as shown in Figure 4.

To avoid any unnecessary debate about the existence per se, we will correctly assume that all chemicals, regardless of their degree of purity, always contain some proportion of an impurity. In fact, the *thresholds* mentioned in Figure 3 support the notion that there is no 100% pure chemical, and we can therefore assume that “presence” in a metaphysical sense is confirmed, which again, does not imply that we have any information about this.

We will then need to distinguish three different types of information along three different axes in a Cartesian Space. First and maybe most importantly, to which degree are we informed about the presence of an impurity? Here, analytical chemistry will provide some answers, for instance, by hints from mass balances and odd signals in various spectra, such as MS and NMR. This information about the presence of an impurity along the x-axis is gradual, and increases, for instance, if more and more “odd” signals in other methods such as Atomic Absorption Spectroscopy (AAS) or TLC support the notion that “there is something impure in there”. The point for the given impurity moves, therefore, in the Space by time along the x-axis.

Secondly, is information about chemical structure or chemical identity of the impurity available? Here we may have a mass peak, a spectrum, an elemental composition, an electronic structure, or even information about optical isomers. This information tends to increase as chemical analysis is more refined and may also explore additional aspects, such as structural specifics. The information continues to increase, and hence the impurity in question moves smoothly along the y-axis.

The z-axis is crucial for pharmacy and relates to the information available regarding biological activity of the impurity. Does the impurity result in any harm or adverse effects to humans within its degree of presence? Or could it harm the environment if and when metabolized and excreted? Information along this dimension is often limited, as the Registration, Evaluation, Authorisation, and Restriction of Chemicals (REACH) exercises on the toxicity of chemicals have demonstrated. Still, such information about an impurity can be collected and may increase by time. The pinpoint of the impurity can therefore move along the z-axis [30].

Within a perfect scenario, the impurity resides in the corner of maximal or sufficient information in all three directions (i.e., about presence, the chemical identity, and the biological activity of the impurity). Its triplet of spatial coordinates is maximal, as defined by the amount of information we agree as necessary to satisfy the safety of the drug. Such an impurity is now established, just as the sylvite impurity in the cooking salt example, or the hair swimming on top of the sausage soup, both occupying position (X_n_,Y_n_,Z_n_), with n being set by convention as “maximal” for the moment—and certainly open to expansion as analytics and regulatory requirements advance. In fact, n for cooking salt is bound to increase these days as the microplastic issue turns into a major issue.

**Figure 5:**
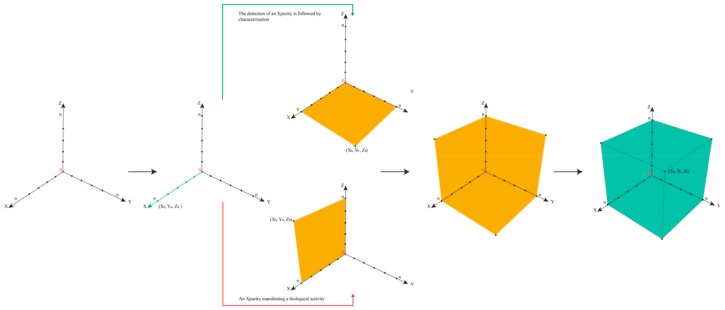
Illustration of the location of an Xpurity within the Cartesian Space of Information. After the detection of an Xpurity in a pharmaceutical product, three major scenarios are possible. The first would concur with the risk management approach and assume that since the presence of this Xpurity is below the identification threshold, it constitutes no danger for patients. The second would be the risk-free option of characterizing this Xpurity regardless of a threshold as indicated by the green arrow. The third scenario, indicated by the red arrow, represents a devastating event (i.e., the presence of a toxic Xpurity manifesting itself by serious side effects in patients). The transition from the state of gathering information i.e., the presence, chemical structure and biological activity about an Xpurity, is represented by the Space of Information changing its colour from yellow to green.

Any impurity not residing in this corner of maximum X_max_, Y_max_, and Z_max_ is problematic, for different individual reasons. Table 2 illustrates the eight epistemological and logically possible scenarios of an impurity in a drug sample or drug product. The first scenario is the most problematic, as an Xpurity escapes detection entirely, Figure 5. One example for (X_0_,Y_0_,Z_0_) is provided by the historical developments surrounding Agent Orange. Its toxic impurities started at (X_0_,Y_0_,Z_0_), then moved up along z as the adverse health impact was noted, and after years of analysis turned “chemistry” along the y-axis with the discovery of the chemical structures of the impurities.

Another particularly nasty situation is faced in the case of impurities manifesting their presence on the impurity profiles of substances and being responsible for devastating side effects although still lacking “chemistry”. This case of (X_n_,Y_0_,Z_n_) is reflected by the infamous impurity Peak X in L-tryptophan, which has long puzzled manufacturers [31]. This structurally unidentified substance represented by a minor chromatographic peak in fact caused an outbreak of eosinophilia–myalgia syndrome in the late 1980s [32]. Efforts were successful in 2003 to conclude that Peak X was as the putative neurotoxin Trp-4,5D [33].

The situations of RbCl and CsCl in the cooking salt, the hidden hair, and the NDMA and NDEA in Valsartan and Ranitidine differ, as we are faced here with impurities of established chemistry and biology. This case of (X_0_,Y_n_,Z_n_) is almost the opposite of the contaminated tryptophan case, yet it is equally nasty and frequently discussed in pharmaceutical and food scandals as the presence of such impurities alludes to an ethical dimension. As we have mentioned already, this situation depends heavily on the manufacturer and manufacturing processes (e.g., uncontrolled reaction conditions and contaminated equipment) and is sometimes due to suboptimal synthesis conditions provided by the most “economical” producers. Causes of such contaminations can be manifold, from chemical side products to abrasion, environmental droppings, and dirty tools. This category designated by (X_0_,Y_n_,Z_n_) also includes the asbestos impurity recently found in baby powder (i.e., an impurity generated during the mining process of talcum and object of a serious lawsuit against Johnson and Johnson in 2017) [34,35,36].

Both of these cases (i.e., nitrosamines and asbestos) need to be distinguished from (X_n_,Y_n_,Z_0_). Again, this case is common in pharmacy as it relates to chemicals lacking a biological profile. REACH has been mentioned already, but most chemicals have not been evaluated fully for their biological impact. In fact, any novel compound synthesized starts at this point, and although its presence and chemical structure may have been established, there is almost unavoidably a gap in biological information.

Lead in petrol, for instance, had long been considered unproblematic, until its damage to the environment was noted and it was phased out in the 1980s [37,38]. Similarly, sulphanilamide elixir was sold in the 1930s, containing the solvent impurity diethylene glycol. Although the presence and the chemistry of this impurity could or should have been clear based on the manufacturing process, the biological danger was ignored until the first patients fell seriously ill [39].

**Figure 6:**
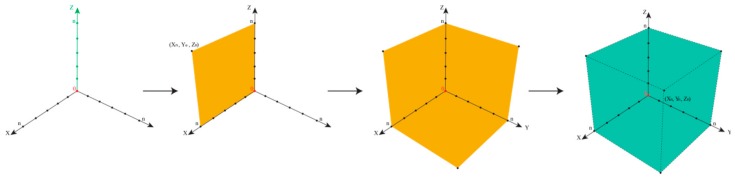
Illustration of dioxin impurity in Agent Orange (see text for details)**.**

**Figure 7:**
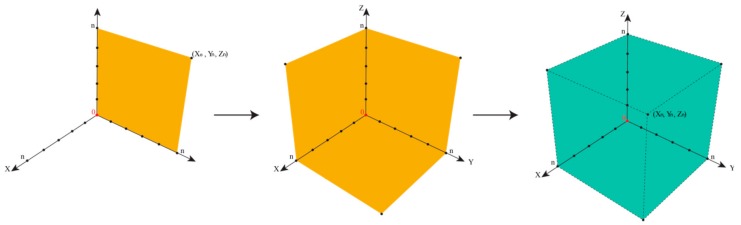
Illustration of NDMA impurity in Valsartan (see text for details).

The other scenarios indicated in Table 2 are less common, yet there are probably examples for each of them. Furthermore, Figure 4 is therefore not only able to distinguish between different kinds of scenarios related to impurities, it is also a versatile tool to place such suspects of modern pharmaceutical scandals into the proper corner and to decide how to proceed (i.e., if any action, analytical or otherwise, needs to be taken). The Cartesian Space of Information related to impurities also enables historical trajectories and these are highly instructive for research and for discussing cases with similar trajectories. Dioxin, for instance, would start in low positions on all coordinates, slowly moving along the z-axis as the toxicity of the herbicide was investigated, and only later move along the x- and then the y-axis, as indicated in Figure 6. In contrast, the NDMA impurity is a compound with high y- and z-values initially, and here the x-axis (i.e., the information about its presence) had been low recently, as discussed already, Figure 7.

There are probably numerous examples which could continue this discussion. Indeed, modern history is, unfortunately, rich with cases of disastrous impurities, although they could have been avoided as in most of these cases, good y- and z-values had been established before. In 1941, Sulfathiazole tablets contaminated with phenobarbital impurities harmed 300 people [40]. Two decades later, Tylenol was tampered with cyanide [41]. In our model, these impurities locate low on the x-axis and high both on the y- and z-axes. They represent the classical case of an unexpected impurity whose occurrence can be averted by expertise and the implementation of proper analytical methods.

Notably, the epistemological approach illustrated in Figure 4 is not limited to Réné Descartes (1596 – 1650) and his space. The triplet Cartesian coordinates (X_n_, Y_n_, Z_n_) are employed here as a start, although an n-tuple can also be constructed by expanding this mathematical model to include other criteria related to the pharmaceutical perspective, such as economic aspect of handling impurities—and indeed this flexibility allows this mathematical model not only to be Cartesian as it may develop further into a “Multidimensional Space of Information”.

## 5. Conclusions

In summary, we have demonstrated that the issue of impurities in medications is complicated and involves aspects of chemistry, biology, drug safety, and analytical power, and also epistemology and ethics. We have also noticed that current regulation must necessarily provide room for “unidentified impurities”, that is, substances which may reside outside the realm of current chemical knowledge. Such substances require a closer inspection, by chemical and epistemological analysis. For this purpose, we have designed a Space of Information which can be employed to illustrate the status of such impurities and also point to possible modes of action. As we have witnessed, we only find what we aim to look for, in general and also in medications. Indeed, the Valsartan scandal of 2018 with its unexpected contaminations has now led authorities to investigate routinely for these impurities in other medications. On the 20 December 2019, the EMA published new quality requirements mandating pharmaceutical manufacturers to perform risk assessments on every chemically synthesised API to determine if nitrosamines are present or not [42].

The Space of Information is therefore a versatile tool to pinpoint impurities despite their “hidden agenda”, to nail down Xpurities, and to explain certain historical and present-day scandals in pharmacy connected with impurities. For pharmacists, it should provide a continued impetus for pharmaceutical analysis and research and may also be refined and adjusted to include additional aspects and categories of drug safety and research.

## Figures and Tables

**Table 1:** Some of the most commonly employed analytical methods in the pharmaceutical industry.

Separation and Isolation Methods	Spectroscopic Methods
capillary electrophoresis, solid-phase extraction, liquid–liquid extraction, accelerated solvent extraction, supercritical fluid extraction, gas/high-pressure liquid/supercritical fluid/thin-layer/high-performance thin-layer/column/flash chromatography	ultraviolet spectrophotometry, infrared spectrophotometry, Raman spectroscopy, mass spectrometry, and nuclear magnetic resonance spectroscopy

**Table 2:** Possible scenarios of an impurity in a drug sample or drug product with insufficient information about either existence, chemistry, or biology. Grey denotes to the variables associated with missing information.

Presence	Chemical Identity	Biological Activity
X_0_	Y_0_	Z_0_
X_n_	Y_0_	Z_0_
X_0_	Y_n_	Z_0_
X_0_	Y_0_	Z_n_
X_n_	Y_n_	Z_0_
X_0_	Y_n_	Z_n_
X_n_	Y_0_	Z_n_
X_n_	Y_n_	Z_n_
